# Permanent draft genome of strain ESFC-1: ecological genomics of a newly discovered lineage of filamentous diazotrophic cyanobacteria

**DOI:** 10.1186/s40793-016-0174-6

**Published:** 2016-08-24

**Authors:** R. Craig Everroad, Rhona K. Stuart, Brad M. Bebout, Angela M. Detweiler, Jackson Z. Lee, Dagmar Woebken, Leslie Prufert-Bebout, Jennifer Pett-Ridge

**Affiliations:** 1 Exobiology Branch, NASA Ames Research Center, Moffett Field, CA USA; 2 Bay Area Environmental Research Institute, Petaluma, CA USA; 3 Physical and Life Sciences Directorate, Lawrence Livermore National Laboratory, Livermore, CA USA; 4 Current address: Division of Microbial Ecology, Department of Microbiology and Ecosystem Science, Research Network “Chemistry meets Microbiology”, University of Vienna, Vienna, Austria

**Keywords:** Cyanobacteria, Nitrogen fixation, Hydrogenase, Intertidal microbial mat

## Abstract

The nonheterocystous filamentous cyanobacterium, strain ESFC-1, is a recently described member of the order *Oscillatoriales* within the *Cyanobacteria*. ESFC-1 has been shown to be a major diazotroph in the intertidal microbial mat system at Elkhorn Slough, CA, USA. Based on phylogenetic analyses of the 16S RNA gene, ESFC-1 appears to belong to a unique, genus-level divergence; the draft genome sequence of this strain has now been determined. Here we report features of this genome as they relate to the ecological functions and capabilities of strain ESFC-1. The 5,632,035 bp genome sequence encodes 4914 protein-coding genes and 92 RNA genes. One striking feature of this cyanobacterium is the apparent lack of either uptake or bi-directional hydrogenases typically expected within a diazotroph. Additionally, a large genomic island is found that contains numerous low GC-content genes and genes related to extracellular polysaccharide production and cell wall synthesis and maintenance.

## Introduction

Microbial mats played a key role in the evolution of the early Earth and today provide a model system for exploring relationships between evolution, ecology, and biogeochemical cycles. In many mats, nitrogen-fixing filamentous *Cyanobacteria* are often central components with important roles in carbon, nitrogen and sulfur cycling [1, 2]. Recently, a previously unknown lineage of filamentous nitrogen-fixing *Cyanobacteria* was described in intertidal microbial mats from Elkhorn Slough, Moss Landing, California [3]. The type strain of this organism, ESFC-1, lacks both heterocysts and an extracellular sheath and has been shown to be an important cyanobacterial diazotroph in the Elkhorn Slough system [3]. At Elkhorn Slough this strain is often a dominant cyanobacterial member of the community (along with *Cyanobacteria* closely related to *Coleofasciculus chthonoplastes*PCC 7420); the sequence abundance of ESFC-1 in 16S rRNA libraries based on DNA and cDNA has been observed to reach up to 5 % (based on pyrosequencing) and 33–36 % (based on clone libraries and pyrosequencing), respectively [3, 4]. Although it is not always dominant, ESFC-1 is highly active, based on *nifH* transcript abundance and rRNA transcript to rRNA gene ratios [3, 5]. Recent work has shown that ESFC-1 produces a considerable external carbon pool as an EPS; this EPS is managed by means of an active exoproteome, and provides a source of organic carbon for the cyanobacterium and other community members [6]. Previous phenetic analyses using full-length 16S rRNA gene sequences have indicated this organism shares only a moderate identity with other identified *Cyanobacteria*; its best cultured BLAST hit is the marine *Aphanocapsa* sp. HBC6 at 93.6 % similarity [7, 8]. Given the importance of ESFC-1 in the Elkhorn Slough mat system and its evolutionarily divergent 16S rRNA, the genomic sequence was determined [3, 8]. Here we report a detailed description of the genome of ESFC-1 as it relates to the ecology of this important mat community organism.

## Organism information

### Classification and features

**Fig. 1:**
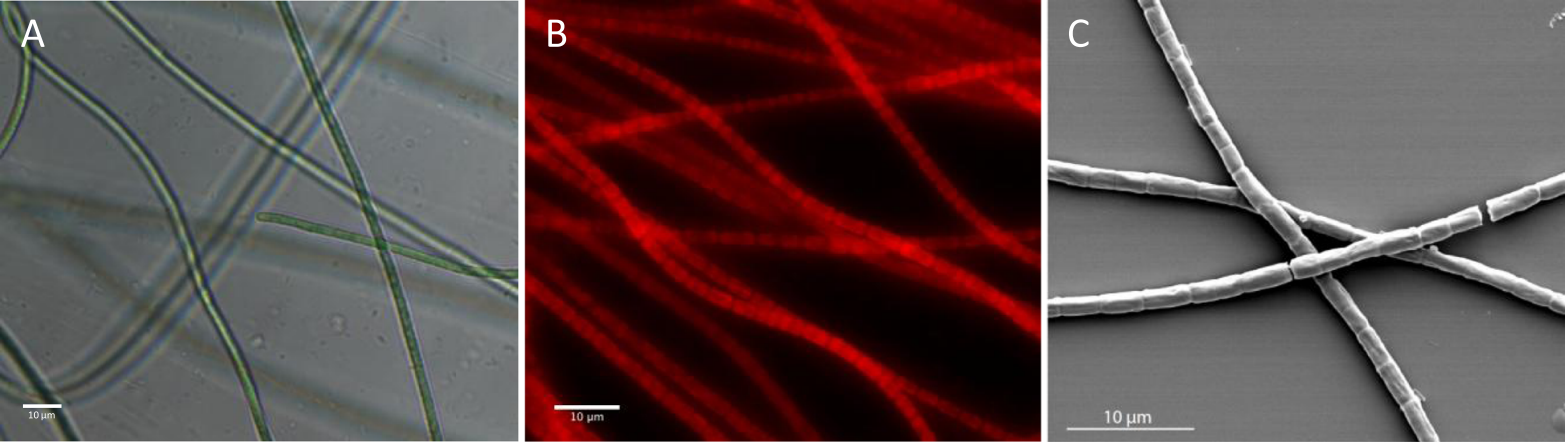
**a** Photomicrograph (400×) showing the filament morphology and size of *Cyanobacterium* ESFC-1, **b** Epifluorescent image of an ESFC-1 biofilm. Image is a calculated maximum intensity projection of a 50 μm z-stack. *Red* is autofluorescent ESFC-1 trichomes. Cells were fixed with 10 % formaldehyde prior to imaging. **c** Scanning electron microscopy image of ESFC-1 trichomes. ESFC-1 samples were fixed with 10 % formaldehyde, rinsed with sterile water, spotted onto a silicon wafer, air-dried and coated with ~5 nm of gold. Imaged with an FEI Inspect F SEM (Hillsboro, OR). For all panels, scale bar represents 10 μm

Strain ESFC-1 was isolated by L. Prufert-Bebout at NASA Ames Research Center in Moffett Field, California from the top 2 mm of intertidal microbial mat samples collected at Elkhorn Slough, California, USA. Fresh microbial mat was repeatedly streaked onto plates of a modified version of ASN artificial seawater medium, until a unialgal culture was obtained [3, 9]. Strain ESFC-1 is a motile, Gram negative, non-heterocystous filament (Fig. 1). Trichomes are cylindrical in shape and straight to slightly curved, with rounded to slightly conical ends. Individual cells are approximately 1.8 μm across, and cells are typically longer than wide, up to 3.5 μm in length, slightly longer than reported previously [3]. Constrictions between cells are shallow but clearly visible. Hormogonia and akinetes have not been observed. Heterocysts have not been observed in cells, even when actively fixing N_2_. Morphologically, ESFC-1 appears most similar to isolates of the form-genus *Geitlerinema*, but with a cell size more typical of the form-genus *Leptolyngbya* [10].

**Fig. 2:**
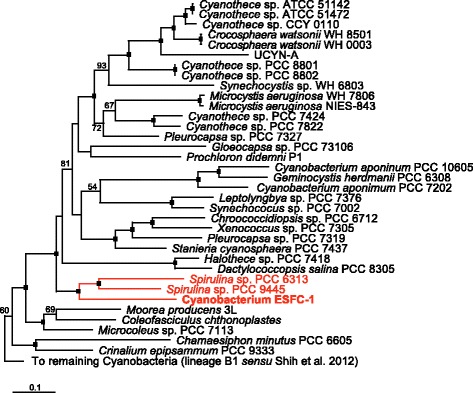
Maximum likelihood (ML) phylogenomic analysis of the cyanobacterial radiation based on a concatenated amino acid sequences for 31 conserved loci [35, 42] showing the phylogenomic affiliation of ESFC-1 with two species of *Spirulina* (PCC 6313 and PCC 9445; clade in *red*). Only the portion of the larger tree corresponding to lineage B2 (*sensu* [42]) is shown. The full 126-taxon ML tree was built using PHYML using the LG protein substitution matrix, and was rooted with *Chloroflexus auranticus* J-10, *Rhodobacter sphaeroides* 2.4.1, *Heliobacterium modesticaldum* Ice1, and *Chlorobium tepidum* TLS [43–45]. ML bootstrap values for nodes >50 are shown; *black boxes* at the nodes denote bootstrap values of 100. Strain ESFC-1 and the nearest neighbors are highlighted in *red*

General features of ESFC-1 and project information are presented in Tables 1 and 2. In previous similarity and phylogenetic analyses based on the 16S rRNA locus, strain ESFC-1 did not show a close similarity (<94 %) with any other cyanobacterial sequence, and its phylogenetic placement within the cyanobacterial radiation was ambiguous [3, 8]. A 31-marker gene phylogenomic analysis of the cyanobacterial radiation, including ESFC-1, is presented in Fig. 2. This analysis places ESFC-1 with strong support in a clade with two non-diazotrophic *Spirulina* strains, PCC 6313 and PCC 9445 [10].

**Table 1:** Classification and general features of strain ESFC-1 according to the MIGS recommendations [38]

MIGS ID	Property	Term	Evidence code^a^
	Classification	Domain *Bacteria*	TAS [39]
		Phylum *Cyanobacteria*	TAS [40]
		Class *Cyanobacteria*	TAS [10]
		Order *Oscillatoriales*	TAS [10]
		Family *Oscillatoriaceae*	TAS [10]
		Genus Unclassified	TAS [3]
		Species Unclassified	TAS [3]
		strain: ESFC-1	
	Gram stain	Negative	NAS
	Cell shape	Cylindrical cells in filaments	TAS [3]
	Motility	Motile	IDA
	Sporulation	Not reported	NAS
	Temperature range	Mesophilic	NAS
	Optimum temperature	Unknown	NAS
	pH range; Optimum	Unknown	NAS
	Carbon source	Photoautotroph	TAS [3]
MIGS-6	Habitat	Marine/Intertidal	TAS [3]
MIGS-6.3	Salinity	Euryhaline; 3.5 % NaCl (w/v)	TAS [3]
MIGS-22	Oxygen requirement	Aerobic	NAS
MIGS-15	Biotic relationship	free-living	TAS [3]
MIGS-14	Pathogenicity	Non-pathogenic	NAS
MIGS-4	Geographic location	Elkhorn Slough, California, USA	TAS [3]
MIGS-5	Sample collection	October, 2009	TAS [3]
MIGS-4.1	Latitude	36°48′47″N	TAS [3]
MIGS-4.2	Longitude	121°47′5″W	TAS [3]
MIGS-4.4	Altitude	1.5 m	TAS [3]

^a^Evidence codes - IDA: Inferred from Direct Assay; TAS: Traceable Author Statement (i.e., a direct report exists in the literature); NAS: Non-traceable Author Statement (i.e., not directly ob-served for the living, isolated sample, but based on a generally accepted property for the species, or anecdotal evidence). These evidence codes are from the Gene Ontology project [41]

**Table 2:** Project information

MIGS ID	Property	Term
MIGS-31	Finishing quality	Improved High-Quality Draft
MIGS-28	Libraries used	Two Illumina PE libraries: 222 bp avg. insert, and 7791 bp avg. insert
MIGS-29	Sequencing platforms	Illumina
MIGS-31.2	Fold coverage	719×
MIGS-30	Assemblers	Velvet v. 1.1.05; ALLPATHS v. r38445; Phrap v. 4.24
MIGS-32	Gene calling method	Prodigal 2.5
	Genbank ID	ARCP00000000
	Genbank Date of Release	December 25, 2014
	GOLD ID	Gi14129
	BIOPROJECT	PRJNA165547
	NCBI taxon ID	1128427
MIGS-13	Source Material Identifier	ESFC-1
	Project relevance	Cyanobacterial ecology

## Genome sequencing information

### Genome project history

Strain ESFC-1 was selected for sequencing because of its recent discovery as a major diazotroph in intertidal mat communities and its unique taxonomic position within the cyanobacteria. The genome project is deposited in the Genome On Line Database (GOLD Legacy ID Gi14129) and the complete genome sequence is deposited in GenBank (accession ARCP00000000). Sequencing, finishing and annotation were performed by the DOE-JGI. A summary of the project information is shown in Table 2.

### Growth conditions and genomic DNA preparation

ESFC-1 was maintained in culture in liquid modified ASN at 25 °C on a 14:10 L:D cycle under cool fluorescent lamps at approximately 50 μmol photons · m^-2^ · s^-1^. High molecular weight genomic DNA was isolated based on the “JGI Bacterial DNA isolation CTAB” protocol from JGI [11], including an RNA digestion step according to the protocol. 50 μg of gDNA was provided to the JGI for sequencing.

### Genome sequencing and assembly

The high-quality draft genome of strain ESFC-1 was generated by the DOE-JGI using the Illumina GAIIx platform [12]. An Illumina standard short-insert paired-end library with an average insert size of 222 bp +/− 50 bp generated 15,283,374 reads. An Illumina CLIP-PE long-insert paired-end library with an average insert size of 7791 +/− 660 bp generated 18,062,354 reads [13]. In total, 4099 Mbp of Ilumina data were generated.

The Illumina draft data was assembled with Allpaths, version r38445 [14], and contained 117 contigs in 14 scaffolds. The consensus was computationally shredded into 10 Kbp overlapping fake reads (shreds). The draft data was also assembled with Velvet, version 1.1.05 [15], and the consensus sequences were computationally shredded into 1.5 Kbp overlapping shreds. The Illumina draft data was reassembled with Velvet using the shreds from the first Velvet assembly to guide the reassembly. The consensus from this second Velvet assembly was shredded into 1.5 Kbp overlapping fake reads. Fake reads from the Allpaths and both Velvet assemblies were assembled using parallel phrap, version 4.24 (High Performance Software, LLC) with a subset of the Illumina CLIP-PE reads [16, 17]. Possible misassemblies were checked and manually corrected in Consed [18]. Gap closure was accomplished using repeat resolution software (Wei Gu, unpublished). The final assembly is based on 4099 Mbp of Illumina draft data, with an average genome coverage of 719×.

### Genome annotation

The genome was annotated automatically with Prodigal 2.5 [19] by IMG [20], locally using the RAST server [21], and by GeneMarkS+ [22] by the NCBI annotation pipeline. Pathways of interest were mapped to the KEGG maps through both IMG and RAST.

## Genome properties

**Fig. 3:**
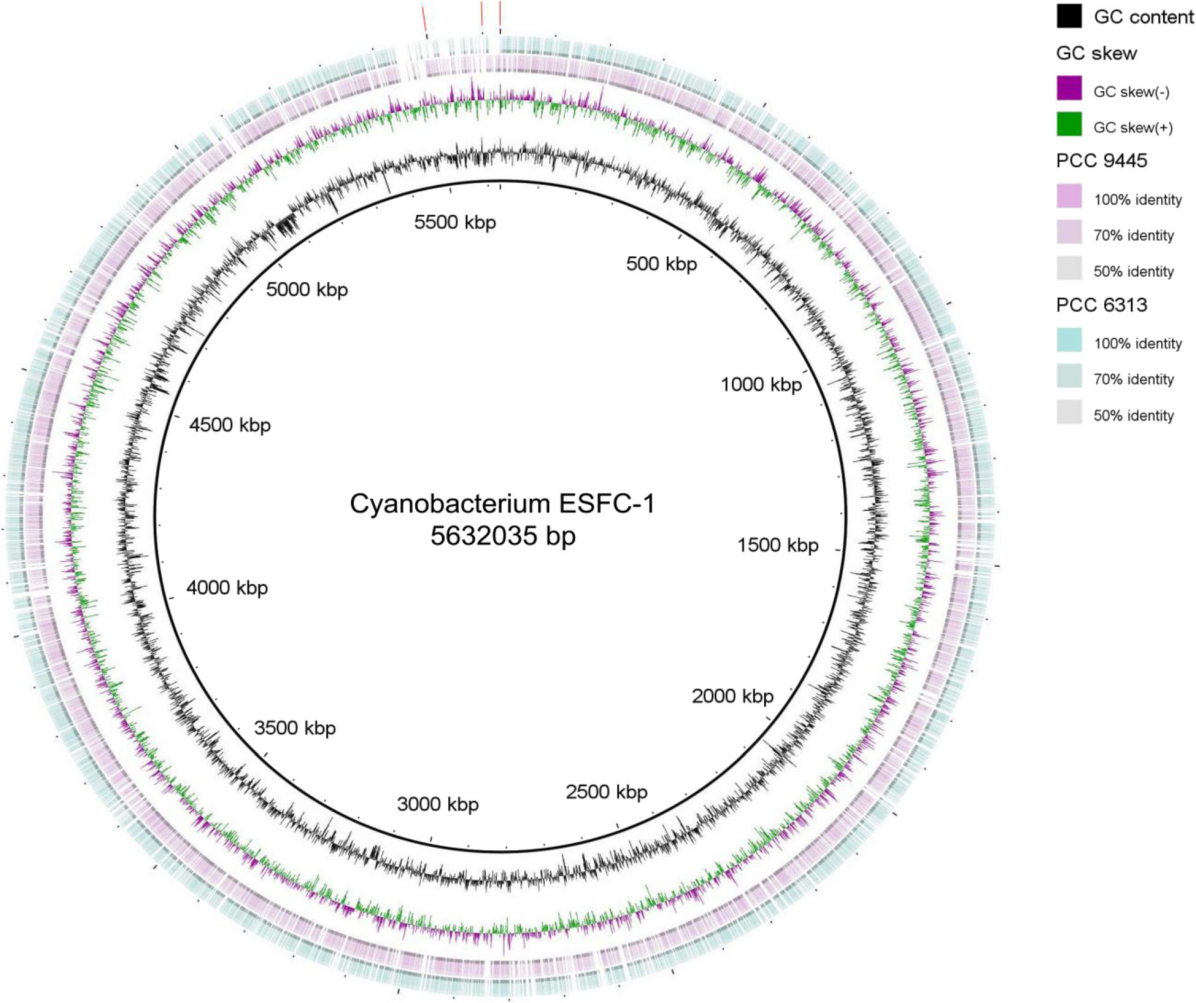
Circularized representation of the three contigs of the genome of strain ESFC-1, with comparisons to *Spirulina* spp. PCC 9445 and PCC 6313, the closest relatives to ESFC-1 with genomes available. From center to outside: GC content, GC skew, genes on forward strand, genes on reverse strand. *Red lines* denote the location of the three contigs in this map. The identified genomic island begins at approximately 5050 kbp. Map was generated by BRIG [46]

The high quality draft genome of cyanobacterium ESFC-1 was resolved to 3 scaffolds consisting of 5,431,811, 135,349 and 64,875 bp, for a total of 5,632,035 bp. GC content was 46.47 %. The genome sequence is predicted to encode 5006 total genes, with 92 RNA genes, and 4914 protein-encoding genes. A majority (79.0 %) of genes were assigned putative functions, and the remainder were annotated as hypothetical proteins. The properties of the ESFC-1 genome, and the distribution of genes into COG functional groups are presented in Tables 3, 4, and Fig. 3. 16S rRNA gene sequence similarity to closely related cultured cyanobacteria, as determined by the phylogeny in Fig. 2, is summarized in Table 5 for the two 16S rRNA genes found in this genome.

**Table 3:** Genome statistics

Attribute	Value	% of total
Genome size (bp)	5,632,035	100.00
DNA coding (bp)	4,742,170	84.20
DNA G+C (bp)	2,617,048	46.47
DNA scaffolds	3	100.00
Total genes	5006	100.00
Protein coding genes	4914	98.16
RNA genes	92	1.84
Pseudo genes	146	2.92
Genes in internal clusters	880	17.58
Genes with function prediction	3505	70.02
Genes assigned to COGs	2625	52.44
Genes with Pfam domains	3742	74.75
Genes with signal peptides	253	5.05
Genes with transmembrane helices	1157	23.11
CRISPR repeats	19	0.25

**Table 4:** Number of genes associated with the general COG functional categories

Code	Value	% of total (2895)	Description
J	194	6.70	Translation, ribosomal structure and biogenesis
A	0	0.00	RNA processing and modification
K	106	3.66	Transcription
L	94	3.25	Replication, recombination and repair
B	2	0.07	Chromatin structure and dynamics
D	27	0.93	Cell cycle control, cell division, chromosome partitioning
Y	0	0.00	Nuclear Structure
V	96	3.32	Defense mechanisms
T	230	7.94	Signal transduction mechanisms
M	223	7.70	Cell wall/membrane biogenesis
N	37	1.28	Cell motility
Z	0	0.00	Cytoskeleton
W	12	0.41	Extracellular Structures
U	33	1.14	Intracellular trafficking and secretion
O	145	5.01	Posttranslational modification, protein turnover, chaperones
C	139	4.8	Energy production and conversion
G	143	4.94	Carbohydrate transport and metabolism
E	201	6.94	Amino acid transport and metabolism
F	73	2.52	Nucleotide transport and metabolism
H	187	6.46	Coenzyme transport and metabolism
I	74	2.56	Lipid transport and metabolism
P	167	5.77	Inorganic ion transport and metabolism
Q	51	1.76	Secondary metabolites biosynthesis, transport and catabolism
R	398	13.75	General function prediction only
S	205	7.08	Function unknown
-	2381	47.56	Not in COGs

**Table 5:** Near full-length 16S rRNA gene similarity matrix for ESFC-1, the two most-closely related cyanobacteria *Spirulina* spp. PCC 6313 and PCC 9445 (based on the phylogeny in Fig. 2), and the highest-scoring BLAST hit from the NCBI database (*Aphanocapsa* sp. HBC6, accession EU249123)

	% Identities					
Sequence	ESFC-1 01	ESFC-1 02	PCC 9445	PCC 6313 01	PCC 6313 02	EU249123
ESFC-1 01	1	0.984	0.899	0.905	0.906	0.923
ESFC-1 02		1	0.911	0.917	0.919	0.935
PCC 9445			1	0.915	0.916	0.913
PCC 6313 01				1	0.998	0.922
PCC 6313 02					1	0.923
EU249123						1

## Insights from the genome sequence

ESFC-1 is a nitrogen-fixing cyanobacterium [3]; all structural genes for nitrogenase were detected (*nifHDK* operon; A3MYDRAFT_2398-2400), as were genes required for uptake of nitrate and reduction to ammonia (*narB* and *nirA*; A3MYDRAFT_3316 and A3MYDRAFT_3311, respectively). However, ESFC-1’s sister taxa, *Spirulina*PCC 6313 and PCC 9445, as determined by the phylogenomic analysis presented in Fig. 2, both lack the *nif* operon. Of the cyanobacterial taxa found in the phylogenomic tree, the highest scoring BLAST hit to the translated *nifH* sequence of ESFC-1 belongs to *Halothece* sp. PCC 7418 (second best score overall, 87 % amino acid identity, E-value 0.0). ESFC-1 has homologs required for assimilatory sulfur reduction, and a homolog for a sulfide:quinine oxidoreductase, suggesting it can utilize hydrogen sulfide as an electron donor to photosystem I; a useful trait in mat environments that may periodically become anoxic/sulfidic [23, 24].

ESFC-1 has a full complement of homologs for photosystems I and II, the cytochrome b_6_f complex, photosynthetic electron transport and ATP synthesis. Detected phycobilisome gene homologs indicate a phycocyanin-rich genotype, with phycocyanin, allophycocyanin core, and linker peptide homologs present. ESFC-1 appears to lack the ability to chromatically adapt; phycoerythrin and phycoerythrocyanin genes appear to be absent. A single set of phycocyanin homologs are present (*cpcBA*; A3MYDRAFT_2965-2964).

Complete sets of genes were detected for the Calvin-Benson cycle, the pentose phosphate pathway, Entner-Doudoroff pathway and glycolysis/gluconeogenesis. TCA cycle and the carbon dioxide concentrating mechanism genes were detected, with 18 *hat*/*hatR* homologs found. One locus, for malate dehydrogenase (EC 1.1.1.37) was not detected, however, a putative low-identity alternative protein homolog was found. Strain ESFC-1 contains the necessary gene homologs for most of the lactate and mixed acid fermentation pathways.

## Extended insights

*Cyanobacterium* ESFC-1 appears to lack either a functional uptake or bi-directional hydrogenase. Neither the JGI nor RAST annotations detected these sequences. Extensive manual searches for the *hox* cluster genes (*hoxEFUYH*), encoding the bi-directional hydrogenase, and the *hupSL* genes encoding the uptake hydrogenase commonly found in N-fixing cyanobacteria, were unsuccessful. Similarly, the hydrogenase-maturation enzymes *hypFCDE* were not found, although the *hypAB* locus was detected (A3MYDRAFT_0781-0782). Comparative analyses of strain ESFC-1 with the two closely-related *Spirulina* strains revealed they both lack *nif* and *hupL*, but unlike ESFC-1, both possess *hupS* homologs and the *hox* operon. The *hypFCDEAB* homologs were found dispersed throughout their genomes; a comparative blastp analysis between the *Spirulina*PCC 6313*hox* operon and best hits within strain ESFC-1 did not reveal any evidence of synteny for nearby loci.

The genome of ESFC-1 contains an approximately 56 kbp region of low GC content, with several putatively horizontally transferred ORFs (Fig. 3). Based on the IMG annotation, 51 ORFs were identified (A3MYDRAFT_4511 – A3MYDRAFT_4561), with a global GC content of 39.67 % and individual GC content for loci ranging from 29 to 47 %, compared with the global genome GC content of 46.47 %. At the phylum level and higher, IMG designated 112 ORFs as putatively horizontally transferred within the entire genome; of these 16, or 14.2 %, were found within this island (11 from *Proteobacteria*, and one each from *Bacteroidetes*, *Chloroflexi*, *Deferribacteres*, *Firmicutes* and *Nitrospirae*). Additional blastp analysis against the non-redundant protein database (excluding environmental sequences) for these ORFs revealed 19 have best hits to non-cyanobacterial sequences. Several additional sequences in this island did have best hits to cyanobacterial sequences, but these cyanobacterial homologs appeared to be restricted to ESFC-1 and a few closely-related *Cyanobacteria*. The remaining high-scoring hits for these genes belonged to other bacterial phyla, suggesting that a gene transfer event for these loci into the *Cyanobacteria* occurred in a common ancestor shared by ESFC-1 and the other closely-related *Cyanobacteria*. In total, as many as 32 loci in the island region may have been horizontally transferred, either recently or into an ancestor of ESFC-1. Predicted gene functions for this region are primarily involved with lipopolysaccharide and outer membrane synthesis, including several methyltransferase-like and glycosyltransferase-like enzymes, such as homologs to the *rfaB*, *rfaG*, *rfaS*, loci and to the *rfaL and rfbX* loci related to O-antigen synthesis. Seven of these proteins have been detected via proteomics of an ESFC-1 culture, four of which were extracellular [6], suggesting genes in this island may play a role in extracellular polysaccharide and cell wall synthesis and maintenance. Many *Cyanobacteria* secrete exopolysaccharides with distinct structures and roles, both in protection from stress (ultraviolet radiation, osmotic and metals) and possibly for carbon storage [25]. This genomic island may provide an advantage to ESFC-1 in stress protection, as has been shown with other cyanobacterial genomic islands [26].

## Conclusions

Despite representing a genus-level divergence within the *Cyanobacteria*, based on both 16S rRNA and phylogenomic analyses, the genome of ESFC-1 appears to belong to a typical filamentous cyanobacterium. However, the ESFC-1 genome is striking in its apparent lack of uptake or bi-directional hydrogenases expected within a diazotrophic cyanobacterium.

Although the uptake hydrogenase *hupSL* is found dispersed through the cyanobacterial radiation, to our knowledge, strain ESFC-1 is one of very few N-fixing cyanobacteria to lack this gene [27]. The uptake hydrogenase is generally considered an integral part of the energetically expensive process of N-fixation, allowing the cyanobacterium to recapture hydrogen produced by nitrogenase activity [28]. However, a deficient mutant of *Anabaena* sp. PCC 7120, lacking the large subunit of the uptake hydrogenase, *hupL*, demonstrated similar growth and N-fixation rates compared to the wildtype, but with enhanced hydrogen production under N-fixation conditions [29]. Given this, and the fact that ESFC-1 is known to be an active nitrogen-fixer *in situ* in the Elkhorn Slough intertidal mat community, it appears that some *Cyanobacteria* do not require a classical uptake hydrogenase, yet still perform this critical ecological role.

The bi-directional hydrogenase *hox* is also common within *Cyanobacteria*. It is thought to play roles in fermentation and potentially as an electron valve during photosynthesis to maintain proper redox conditions [30–32]. However, as suggested by Tamagnini et al. [33], the physiological role of the bi-directional hydrogenase in *Cyanobacteria* is unsettled. A recent analysis of 36 cyanobacterial strains indicated that a bi-directional hydrogenase was necessary for hydrogen production via fermentation in these cyanobacteria [34]. Consistent with its apparent lack of a bi-directional hydrogenase, strain ESFC-1 has been shown to produce hydrogen under N-fixation, but not fermentation conditions under laboratory conditions (data not shown). One possible explanation is that strain ESFC-1 ferments under anoxic conditions via lactate or homolactate fermentation pathways, as found within the genome. Such fermentation is known from filamentous *Cyanobacteria*, and allows for maintenance of redox without concomitant production of hydrogen gas [30]. Both *Spirulina* spp. PCC 6313 and PCC 9445 possess homologues for the *hox* genes, so this absence in strain ESFC-1 is best explained by loss, consistent with the uneven distribution of the *hup* and *hox* genes in the cyanobacterial radiation [33].

Finally, since the ESFC-1 genome is not closed, the absent *hox*, *hup* and *hyp* genes are possibly in missing regions, or simply were not detected in the automated annotation process. However, extensive manual searches of the genome failed to find any putative hydrogenases. A search of the draft genome for the 107 marker genes commonly used to estimate completeness in metagenomic analyses found all 107 [35], suggesting the absence of these three gene groups is genuine.

Despite the apparent lack of a functional hydrogenase, strain ESFC-1 has been shown to be a dominant and active member of the Elkhorn Slough community. Further, it appears to be globally distributed. Although this distribution appears more limited compared to the cosmopolitan *C. chthonoplastes*, both *nifH* and 16S rRNA gene environmental sequences similar to ESFC-1 (>95 %) have been observed in the intertidal mats at Guerrero Negro, Mexico [36], and in lake sediments in Daqing, China (unpublished data, accession KJ176902). An isolate, *Leptolynbya* sp. LEGE 07176, from the intertidal zone in Portugal [37] may represent a second isolate of this lineage. As one of the only known N-fixing cyanobacteria natively lacking an uptake hydrogenase, this organism may be a suitable target for hydrogen production research. Future studies of ESFC-1 should experimentally confirm the lack of functioning hydrogenase proteins, and explore the nature and energetics of fermentation and N-fixation, and the ecological consequences for an organism that lacks these key enzymes.

## Abbreviations

CLIP-PE: Cre-LoxP Inverse PCR Paired-End
EPS: Extracellular polymeric matrix
ESFC: Elkhorn Slough filamentous cyanobacterium
